# International normalized ratio and serum C-reactive protein are feasible markers to predict complicated appendicitis

**DOI:** 10.1186/s13017-016-0081-6

**Published:** 2016-06-21

**Authors:** Maru Kim, Sung-Jeep Kim, Hang Joo Cho

**Affiliations:** Department of Trauma Surgery, College of Medicine, The Catholic University of Korea, Seoul, Republic of Korea

**Keywords:** Appendicitis, Complication, C-reactive protein, International normalized ratio, Diagnosis

## Abstract

**Background:**

Diagnostic approach for complicated appendicitis is still controversial. We planned this study to analyze preoperative laboratory markers that may predict complications of appendicitis.

**Methods:**

Patients who underwent appendectomy were retrospectively recruited. They were divided into complicated appendicitis and non-complicated appendicitis groups and their preoperative laboratory results were reviewed.

**Results:**

A total of 234 patients were included. Elevated international normalized ratio (INR) and serum C-reactive protein (CRP) were associated with complicated appendicitis (*p* = 0.001). On ROC curve analysis, area under the curve (AUC) of CRP and INR were 0.796 and 0.723, respectively.

**Conclusions:**

INR and CRP increased significantly in patients with complicated appendicitis. Further studies evaluating INR and CRP in patients undergoing conservative management for appendicitis are required.

## Background

Appendicitis is a common disease. Lifetime risk for having appendicitis is reported to be about 7 to 10 % [1, 2]. Usual management of appendicitis is laparoscopic appendectomy, which has lower complication rate, length of hospital stay, and mortality than open appendectomy [3–5]. However, morbidity or mortality after laparoscopic appendectomy could be happened [6–8].

Many studies analyzing the conservative management of appendicitis have been published. Almost all of these studies looked at managing uncomplicated appendicitis [9–11], including the use of antibiotics in conservative management. No definite method is currently available to distinguish complicated from uncomplicated appendicitis preoperatively, despite the fact that several studies about predicting complicated appendicitis were published [3, 12–15]. In this study, we tried to utilize preoperative laboratory markers to predict whether appendicitis was complicated or not.

## Methods

Institutional review board of Uijeongbu St. Mary’s hospital approved this retrospective study and informed consent was waived. Medical records of patients who underwent appendectomy between February 2014 and September 2014 were reviewed. The following data were collected; general characteristics as sex, and age; initial laboratory results including white blood cell counts (WBC), neutrophil percent, serum total bilirubin (TB), serum C-reactive protein (CRP) and international normalized ratio (INR); and perioperative data as operation record, pathologic report, initial body temperature (BT). Hospital stay, and wound complications—defined as pus-like discharge from wound—were checked, as secondary outcomes, to analyze post-operative outcomes. Pathological confirmation of appendicitis was mandatory for inclusion in the study. Patients with other inflammatory conditions such as diverticulitis, pelvic inflammatory disease, torsion of ovary, small bowel perforation and intussusceptions were excluded. Additionally, patients with missing values or after incidental appendectomy were excluded.

Based on patients’ records, perforated appendicitis, periappendiceal abscess, and peritonitis were regarded as complicated appendicitis (CA), whereas other findings were regarded as non-complicated appendicitis (NA). Upper limits for normal WBC, CRP, TB, and INR were specified at 10 × 10^9^/L, 0.3 mg/dL, 1.2 mg/dL and 1.22, respectively. Body temperature higher than 37.2 °C was regarded as fever. Type of appendicitis and clinical outcomes were correlated to laboratory results.

Independent t-test was used for quantitative analysis and Chi-square and Fisher’s exact test were used for qualitative analysis. P-value of less than 0.05 was regarded as statistically significant. Additionally, receiver operating characteristic (ROC) curve analysis was used to evaluate each marker’s accuracy. SPSS version 17.0 (SPSS Inc. Chicago, IL) was used for statistical analysis.

## Results

The medical records of 258 who underwent appendectomy during the period from February 2014 to September 2014 were reviewed. Only 234 patients were eligible for enrollment in the study. Mean age of the patients was 35.8 ± 18.9 years and number of male patients was 126. Fifty-four patients had complicated appendicitis and 180 patients had uncomplicated appendicitis.

Results of laboratory markers and other basic characteristics were analyzed according to the type of appendicitis. Statistically significant elevations in CRP and INR were identified in CA patients (*p* = 0.001). In addition, they were associated with a high relative risk (relative risk [95 % confidence interval]: 1.291 [1.149–1.452], and 2.059 [1.032–4.108], respectively). On the contrary, no other associations between type of appendicitis and preoperative WBC, neutrophil percent, or TB were identified. Similarly, BT showed no statistically significant association with the type of appendicitis. These results are shown in Table 1.

**Table 1:** Characteristics and analysis of patients according to type of appendicitis

		Non-complicated appendicitis (*n* = 180)	Complicated appendicitis (*n* = 54)	p-value
Gender	Male	92 (51.1 %)	34 (63.0 %)	0.125
	Female	88 (48.9 %)	20 (38.0 %)	
Age in years (mean ± SD)		34.7 ± 17.9	39.5 ± 21.7	0.144
WBC	Normal	44 (24.4 %)	11 (20.4 %)	0.536
	Elevated	136 (75.6 %)	43 (79.6 %)	
Neutrophil percent	Normal	70 (38.9 %)	15 (27.8 %)	0.136
	Elevated	110 (61.1 %)	39 (72.2 %)	
C-reactive protein	Normal	52 (28.9 %)	4 (7.4 %)	0.001
	Elevated	128 (71.1 %)	50 (92.6 %)	
Total bilirubin	Normal	138 (76.7 %)	37 (68.5 %)	0.227
	Elevated	42 (23.3 %)	17 (31.5 %)	
Body temperature	Normal	163 (90.6 %)	50 (92.6 %)	0.79
	Elevated	17 (9.4 %)	4 (7.4 %)	
INR	Normal	175 (97.2 %)	46 (85.2 %)	0.001
	Elevated	5 (2.8 %)	8 (14.8 %)	

*WBC* white blood cell counts, *INR* international normalized ratio, *SD* standard deviation, *n* number of patients

**Fig. 1:**
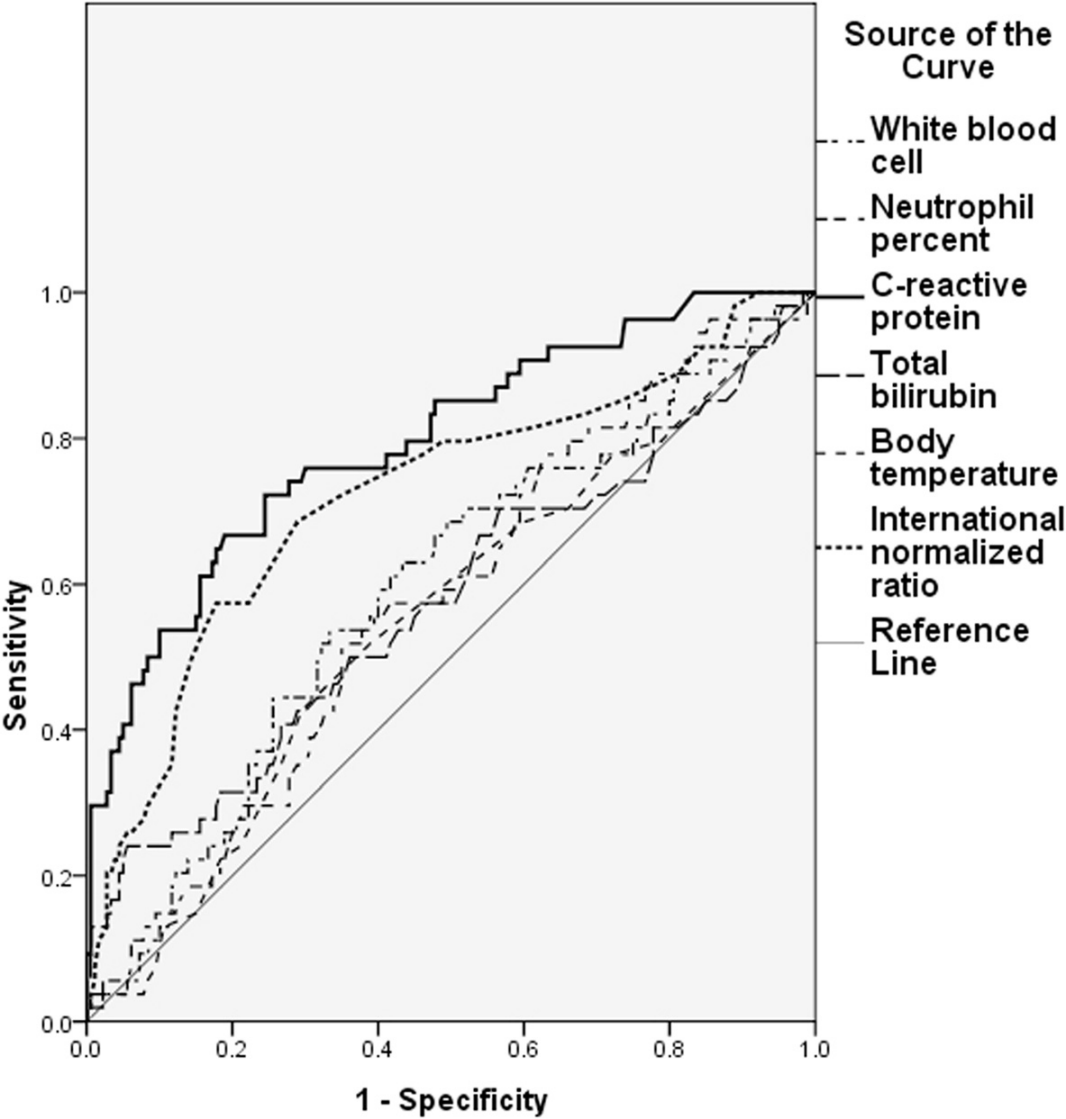
Receiver operating characteristic (ROC) curve for initial laboratory parameters. The area under the ROC curve of CRP and INR was 0.796 and 0.723, respectively

ROC curve analysis was performed to examine the feasibility of each parameter. Area under curve (AUC) of CRP and INR was 0.796 and 0.723, respectively, which showed their feasibility as useful CA predictors. However, AUC of TB was only 0.576, and the other parameters showed similar AUCs that were much lower than those of CRP and INR. Results of ROC curve analysis are summarized in Table 2 and Fig. 1.

**Table 2:** Area under curve from receiver operating curve analysis of each parameter

	Area under curve
WBC	0.594
Neutrophil percent	0.577
C-reactive protein	0.796
Total bilirubin	0.576
Body temperature	0.552
INR	0.723

*WBC* white blood cell counts, *INR* international normalized ratio

Additional analyses of post-operative outcomes, namely hospital stay and wound complications, are summarized in Table 3. Elevated CRP and INR were associated with longer hospital stay (3.20 vs. 3.94 days, *p* < 0.001; 3.70 vs. 4.85 days, *p* = 0.001, respectively). However, they did not show a statistically significant association with wound complication.

**Table 3:** Analysis of clinical outcomes and laboratory findings

		Normal CRP (*n* = 56)	Elevated CRP (*n* = 178)	p-value
Hospital stay (days)		3.20 ± 0.84	3.94 ± 1.29	<0.001
Wound complication	No	47 (83.9 %)	164 (92.1 %)	0.072
	Yes	9 (16.1 %)	14 (7.9 %)	
		Normal INR (*n* = 221)	Elevated INR (*n* = 13)	p-value
Hospital stay (days)		3.70 ± 1.18	4.85 ± 1.68	0.001
Wound complication	No	200 (90.5 %)	11 (84.6 %)	0.072
	Yes	21 (9.5 %)	2 (15.4 %)	

*CRP* serum C-reactive protein, *INR* international normalized ratio, *n* number of patients

## Discussion

Several approaches were proposed to diagnose acute appendicitis. There were many studies about computed tomography (CT) scan in evaluation of acute appendicitis that showed its feasibility as a diagnostic method [16, 17]. However, other studies highlighted the risk from radiation exposure after CT scan [18, 19]. Although the risk of malignancy arising after CT scan is rare and low, however; incorporating CT scan as a routine diagnostic method for appendicitis should be decided carefully, considering the benign nature of the disease.

Many studies looking at the power of CRP in predicting complications of appendicitis have been published recently showing positive results [20, 21]. Similarly, in the present study, elevated CRP was associated with CA and its AUC was the highest among the tested parameters. The results of the present study support the use of CRP as a predictor for complicated appendicitis.

Interestingly, our study focused on the association between INR and type of appendicitis. To the best of our knowledge, this study was the first to look at this association. Our results showed a statistically significant association between INR and both CA as well as postoperative outcome.

Clotting pathway is activated by inflammatory mediators following exposure to infectious agents like viruses and bacteria, or inflammatory cytokines like interleukin-1, interleukin-6, tissue necrosis factors, etc. [22]. Therefore in severe inflammatory conditions as sepsis, patients are prone to have bleeding tendency, known as disseminated intravascular coagulopathy, caused by excessive consumption of coagulation factors [23]. Prothrombin time is the general test used to check bleeding tendency, especially for the extrinsic pathway, and INR represents a mathematical modification of prothrombin time to allow for standardized reporting between different laboratories. We focused on the association between INR and acute appendicitis as we postulated that CA may be associated with bleeding tendency, despite rarely aggravating to sepsis. For preoperative evaluation, INR is a mandatory parameter to check, while CRP is helpful but not essential.

One more point to consider is the cost of each marker. INR costs 2.03 $ per test which is much cheaper than CRP (7.82 $ per test), as per the national health insurance system. Although CRP showed better AUC value, cost-effectiveness should be evaluated in further studies.

We also analyzed post-operative outcomes. Previous studies showed that CA was associated with poorer post-postoperative outcomes [24–26]. Elevated INR and CRP were associated with longer hospital stay; however, no statistical correlation with wound complication was identified. To clarify this association, further studies analyzing postoperative outcome are required.

Additionally, further studies looking at the choice of the management strategy for acute appendicitis according to laboratory findings are required. Proper selection of patients with NA who have normal INR or CRP for conservative management will help to validate conservation, hence avoiding unnecessary appendectomies. Our study may serve as the base for these future studies.

Other limitations of our study include the small sample size and the retrospective single center design. Large-scaled, multicenter-based studies are needed to further elucidate our findings.

## Conclusions

In conclusion, elevated INR and CRP were associated with complicated appendicitis and longer hospital stay. Further studies relating laboratory findings and outcomes of conservative management are required.

## Abbreviations

BT, body temperature; CA, complicated appendicitis; CRP, serum C-reactive protein; INR, international normalized ratio; NA, non-complicated appendicitis; TB, total bilirubin; WBC, white blood cell counts.
